# Supplementary figures and images for: Ciona Brachyury proximal and distal enhancers have different FGF dose-response relationships
Source: PLoS Genet. 2021 Jan 19;17(1):e1009305. doi: 10.1371/journal.pgen.1009305 (PMC7846015; doi:10.1371/journal.pgen.1009305)

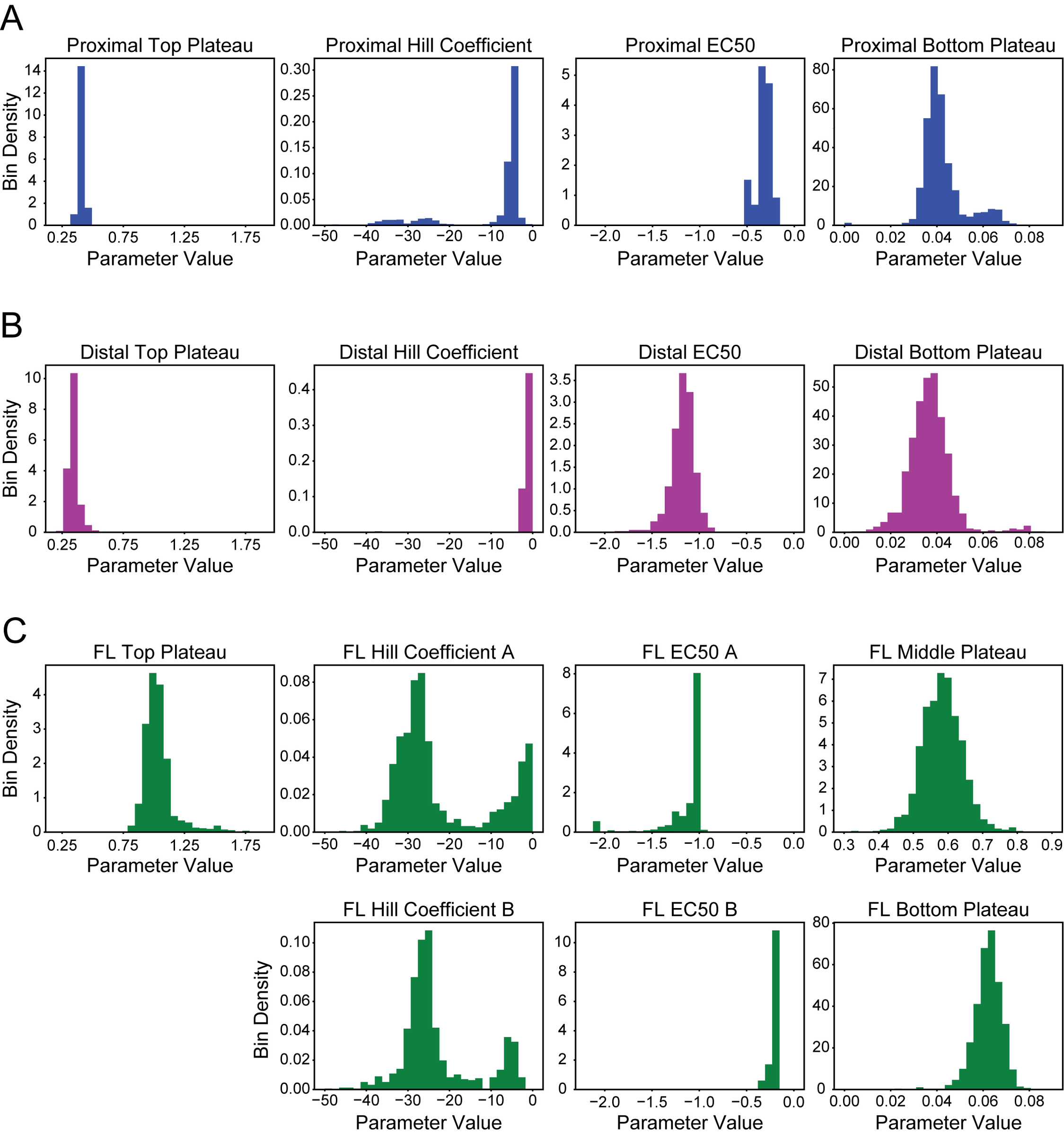

Supplement: S1 Fig — (A-C) Bootstrap parameter distributions for curves fit to the (A) Proximal, (B) Distal, and (C) Full Length whole-embryo reporter data. Monophasic Hill functions are fit for the Proximal and Distal constructs. A biphasic double-sigmoid function is fit for the Full Length construct. (TIF) [file pgen.1009305.s001.tif]

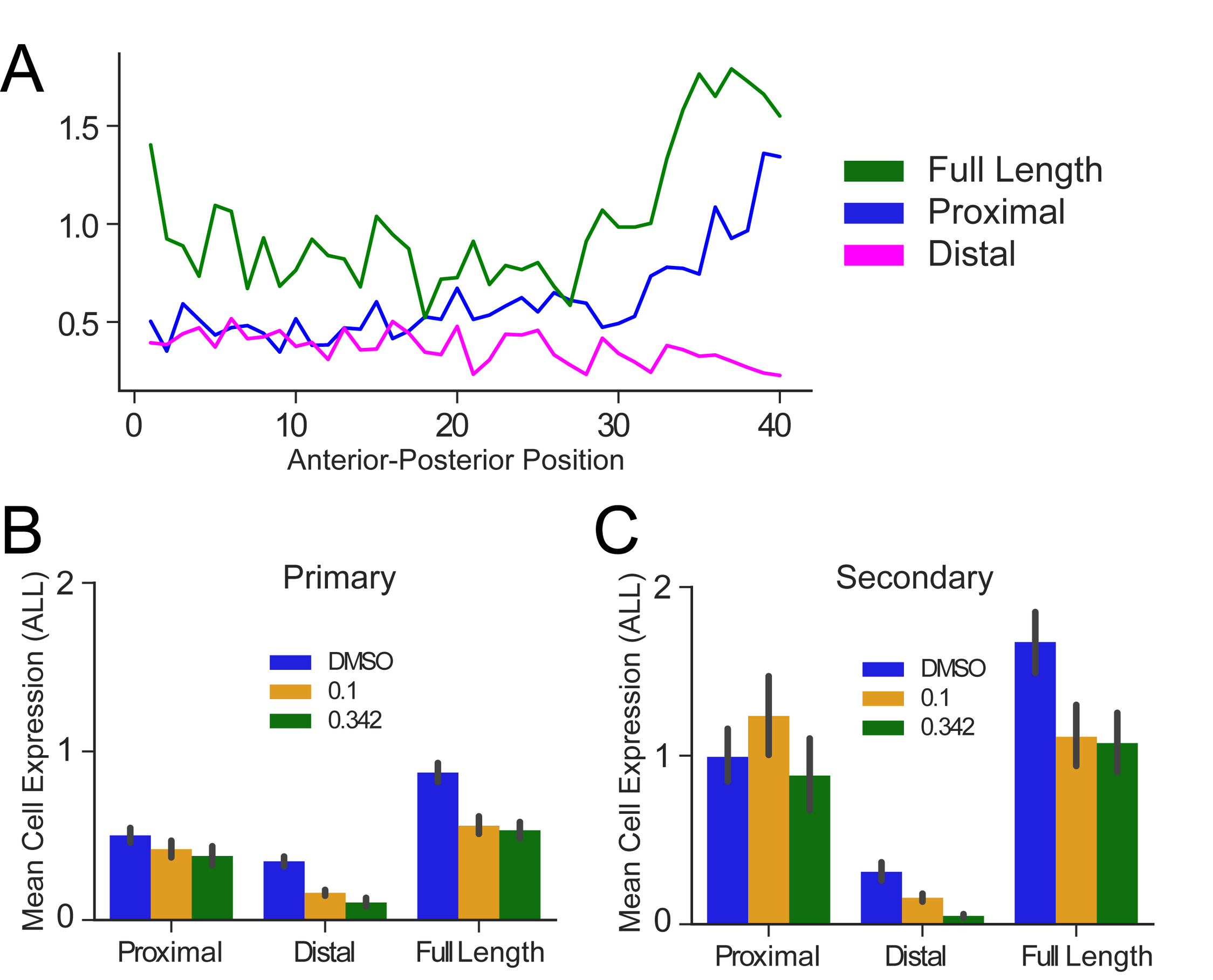

Supplement: S2 Fig — A) Normalized reporter expression as a function of approximate anterior-posterior position in DMSO-treated control embryos. B) Normalized expression of the Proximal, Distal and Full Length reporter constructs at the indicated U0126 doses in primary notochord cells. C) Normalized expression of the Proximal, Distal and Full Length reporter constructs at the indicated U0126 doses in secondary notochord cells. (TIF) [file pgen.1009305.s002.tif]
