# Supplementary material for: Ciona Brachyury proximal and distal enhancers have different FGF dose-response relationships
Source: PLoS Genet. 2021 Jan 19;17(1):e1009305. doi: 10.1371/journal.pgen.1009305 (PMC7846015; doi:10.1371/journal.pgen.1009305)
Supplement: Attachment — Submitted filename: PLOS GENTICS Veeman sept 2020 .pdf [file pgen.1009305.s005.pdf]

This paper compares the responsiveness of two Bra enhancers to FGF signaling and finds that they respond differently to FGF signaling. I enjoyed reading this paper and found the main finding compelling. The techniques and quantification are a novel approach in Ciona.

Concerns:

1. Statements regarding enhancer redundancy
   I disagree with the last statement in the abstract that the different responsiveness to FGF signaling means that these enhancers are fundamentally not redundant elements. My understanding is that redundant enhancers are enhancers with similar expression patterns, sometimes even overlapping but not precisely the same patterns, and that these enhancers can even have different inputs. More importantly, redundancy needs to be tested by removing these enhancers to assay if they are dispensable for normal expression; the paper doesn't test these questions. Thus, although I think it is interesting that the two enhancers have different responses to FGF signaling, I am strongly opposed to the statements relating these findings to enhancer redundancy.
2. Distributed enhancers, why call it this?
   I know this term has been suggested, but I find this misleading as it implies not all expression is within a single unit. I would use **potentially** redundant or shadow enhancers instead.
3. Number of embryos studied.
   While I understand that doing confocal analysis of the embryos limits throughput, I am concerned about the n=10. How were the ten embryos to image chose, was this somehow blinded? I can imagine a situation where picking these embryos could easily bias the results. Is it possible to increase the n? Could you explain how you choose the 10 embryos on the slide to the image? Typically, I would imagine you want to pick the well-developed embryos on a slide, but with the inhibitor's addition, how do you know which embryos to pick? Is this at random? How do you know if the embryos you choose are deformed because of the drug or merely the experimental process?
4. Link between the location of enhancer and response to FGF signaling
   There is some suggestion that the difference in enhancer response to FGF signal is linked to each enhancer's location, proximal vs. distal. I'm not sure if this is intended or just an artifact of how these enhancers are described in the text. Either way, I find this misleading as the location of the enhancers and how this relates to their responsiveness to FGF has not been tested. As the authors later go on to talk about, the difference could be due to enhancer grammar. I think they need to be careful not to imply a link between location and response.
5. Relating in situ to reporter assays
   How are they able to semi-quantitate the in situ? We don't know if the Bra gene is regulated by more than just the two enhancers identified. As a result, it is impossible to relate the Bra endogenous expression and the reporter assays. This should be clearly stated in the text to avoid any confusion or implied comparison between the reporter and the in situ readout.
6. Stating that the enhancers have the same inputs yet respond differently
   Line 343-345. The authors suggest that the two enhancers have the same inputs and yet respond differently to FGF signaling. While in both the enhancers studied, it appears that ETS and Zic are important, the necessity and sufficiency of these features have not been demonstrated. So it is possible that other inputs are also important and indeed that the two enhancers have some differences in inputs. This should be raised as a possibility and not simplified to state that these two enhancers have the same inputs and behave differently.
7. Adding more to the caveats section
   I like the section suggesting caveats, but they should add to this that the necessary and sufficient features within the enhancer have not been experimentally determined and so these enhancers could require other tf binding sites and that there may be differences between the two enhancers and their inputs that have not yet been identified.
