# Supplementary material for: Ciona Brachyury proximal and distal enhancers have different FGF dose-response relationships
Source: PLoS Genet. 2021 Jan 19;17(1):e1009305. doi: 10.1371/journal.pgen.1009305 (PMC7846015; doi:10.1371/journal.pgen.1009305)
Supplement: Attachment — Submitted filename: ShadowEnhancersPaperResponsetoReviews_3.pdf [file pgen.1009305.s006.pdf]

*We thank all three reviewers for their helpful comments and were gratified to see that all three were enthusiastic about the manuscript and in general agreement that our key conclusions are well supported. I believe we have been able to address all of their specific questions and concerns.*

Reviewer #1: The manuscript by Harder et al. 'Ciona Brachyury proximal and distal enhancers have different FGF dose-response relationships' is a very interesting study that convincingly supports the idea that shadow/distributed enhancers are not simply redundant elements. The authors have made use of simple methods (GFP as reporter in transcriptional assays and colorimetric in situ hybridization) to provide quantitative measurements of differential response to Fgf/Erk pathway. In addition, they have shown that endogeneous Brachyury expression has different sensitivities to MAPK inhibition depending on notochord precursors. It would thus be important to determine whether the proximal, distal and 'full length' enhancers also display such differential sensitivity.

*Reviewer 3 also brought up this question about whether the 3 enhancer constructs behave differently in different notochord sublineages. It's a very interesting question but quite difficult to address in terms of identifying modest differences in EC50 like the ones we saw between different notochord founder cells with respect to endogenous Bra expression. The positions of the primary notochord cells become extensively shuffled by intercalation, so the precursor cells can't be reliably inferred at the stages when these reporter constructs have good expression and are most quantifiable. The secondary notochord cells end up at the posterior tip of the tail and can be distinguished from primary cells in wildtype embryos but not at higher doses of U0126. The only way you could really address this question would be to repeat the entire reporter dose/response study in embryos that were cleavage-arrested with cytochalasin at the 112-cell stage before the notochord founder cells finish dividing and intercalate. This would have some of its own caveats, and a considerably larger sample size would probably be needed if you wanted to get good statistics not just on primary versus secondary notochord but also on the 4 distinct primary notochord founder cell pairs. This would potentially be an interesting topic for some future study but is outside the scope of what we can realistically do here.*

*We were able to take an initial look at this question, however, by reanalyzing the data from our single cell quantitation of reporter expression at the 0, 0.1 and 0.342μM doses of U0126. We can't distinguish between the progeny of different primary notochord founder cells in this data but we can identify primary versus secondary with reasonable accuracy. We've included this new analysis as a new supplemental figure S2 and we discuss it in the text at lines 432-476 of the revised manuscript. We find that the proximal and full length constructs have somewhat stronger expression in secondary versus primary notochord cells whereas the distal enhancer if anything tends to be slightly stronger in the anterior primary notochord. We didn't notice striking differences comparing primary versus secondary in terms of expression loss at moderate doses of U0126, but a full dose-response series in cleavage-arrested embryos would really be needed to address this carefully. Secondary cells had very similar dose-response curves to the lateral primary notochord cells in terms of endogenous Bra expression, the reporter assays have much higher variance than the semi-quantitative in situ scores, and the statistical*

*power decreases in some respects as we split cells into different groupings, so we wouldn't necessarily expect to be able to detect sublineage-specific reporter response differences with the data at hand. Given that this new analysis is interesting but somewhat tangential and inconclusive, we thought it made most sense to show it in a supplemental figure.*

Reviewer #2:
This paper compares the responsiveness of two Bra enhancers to FGF signaling and finds that they respond differently to FGF signaling. I enjoyed reading this paper and found the main finding compelling. The techniques and quantification are a novel approach in Ciona.
Concerns:
1. Statements regarding enhancer redundancy I disagree with the last statement in the abstract that the different responsiveness to FGF signaling means that these enhancers are fundamentally not redundant elements. My understanding is that redundant enhancers are enhancers with similar expression patterns, sometimes even overlapping but not precisely the same patterns, and that these enhancers can even have different inputs. More importantly, redundancy needs to be tested by removing these enhancers to assay if they are dispensable for normal expression; the paper doesn't test these questions. Thus, although I think it is interesting that the two enhancers have different responses to FGF signaling, I am strongly opposed to the statements relating these findings to enhancer redundancy.

*We agree with this comment and have changed the last sentence of the abstract to say that they are 'fundamentally not* equivalent *genetic elements' instead of 'fundamentally not* redundant *genetic elements'. We've also added a sentence in the discussion to make it clear that the potential in vivo functional redundancy of these elements has not yet been tested in genetic deletions.*

2. Distributed enhancers, why call it this? I know this term has been suggested, but I find this misleading as it implies not all expression is within a single unit. I would use **potentially** redundant or shadow enhancers instead.

*All of the terms used to describe this phenomenon are somewhat unsatisfactory. I understand this reviewer's concern that 'distributed' might imply a situation where two or more enhancers have to act together in a single transcriptional activation event, but 'shadow' is problematic as well in how it implies a hierarchy of main vs shadow enhancers. It is certainly true, however, that shadow enhancer is the most widely used term and given that this is our first manuscript on this topic, we probably shouldn't be pushing for new terminology. We've changed 'distributed enhancer' to 'shadow enhancer' throughout the revised manuscript and have added a sentence to make it clear that we are not implying a functional hierarchy.*

3. Number of embryos studied. While I understand that doing confocal analysis of the embryos limits throughput, I am concerned about the n=10. How were the ten embryos to image chose, was this somehow blinded? I can imagine a situation where picking these embryos could easily bias the results. Is it possible to increase the n? Could you explain how you choose the 10 embryos on the slide to the image? Typically, I would imagine you want to

pick the well-developed embryos on a slide, but with the inhibitor's addition, how do you know which embryos to pick? Is this at random? How do you know if the embryos you choose are deformed because of the drug or merely the experimental process?

*A larger n within each biological replicate would obviously be desirable, but the confocal imaging really did become limiting given the large number of doses and the three different constructs tested. It is important to note that each dose/reporter combinations was tested across at least three different biological replicates (independent fertilization/ dechorionation/ electroporations), so the sample size ended up being >30 for each dose/reporter combination across the whole study.*

*We were very methodical in how we selected the embryos to image. Some dechorionated Ciona embryos will indeed have non-specific deformations even in the absence of U0126 treatment but these sorts of monster embryos are generally distinguishable from the characteristic phenotypes seen from U0126 treatment. We excluded the obvious non-specific monster embryos but tried to make a random selection from the reasonably representative embryos of each dose. Importantly, the experimenter was completely blind to reporter expression while selecting the embryos to image on each slide. They were selected based solely on embryonic morphology using the phalloidin channel alone. We did not even glance at the reporter channel until the list of embryos to be imaged was finalized and we were acquiring the actual confocal stacks. This was done deliberately to avoid any inadvertent bias in choosing embryos based on reporter expression.*

4. Link between the location of enhancer and response to FGF signaling There is some suggestion that the difference in enhancer response to FGF signal is linked to each enhancer's location, proximal vs. distal. I'm not sure if this is intended or just an artifact of how these enhancers are described in the text. Either way, I find this misleading as the location of the enhancers and how this relates to their responsiveness to FGF has not been tested. As the authors later go on to talk about, the difference could be due to enhancer grammar. I think they need to be careful not to imply a link between location and response.

*We certainly didn't mean to suggest anything about the location of the enhancers being mechanistically important with respect to the quantitative differences in their responses to FGF signaling. It wasn't clear to us exactly what this reviewer found misleading, but we've added a new sentence where we first introduce the different enhancer constructs to make it clear that the 'proximal' and 'distal' language is purely descriptive. We're happy to revisit other language if you point out specific sentences of concern.*

5. Relating in situ to reporter assays How are they able to semi-quantitate the in situ? We don't know if the Bra gene is regulated by more than just the two enhancers identified. As a result, it is impossible to relate the Bra endogenous expression and the reporter assays. This should be clearly stated in the text to avoid any confusion or implied comparison between the reporter and the in situ readout.

*Each cell was subjectively scored on an integer scale from 3 (very strong expression) to 0 (no expression). It's only semi-quantitative, but averaged over many embryos it does give you a nice dose-response curve. We agree that the quantitative reporter dose-response curves and the semi-quantitative in situ dose-response curves can only be superficially compared, and have added a sentence to that effect. We've also added qualifying language about the possibility of other relevant cis-regulatory elements. Subject to those caveats, however, it remains fair to say that the full-length reporter dose-response curve and the semi-quantitative endogenous in situ dose-response curve are at least superficially reminiscent of one another.*

6. Stating that the enhancers have the same inputs yet respond differently Line 343-345. The authors suggest that the two enhancers have the same inputs and yet respond differently to FGF signaling. While in both the enhancers studied, it appears that ETS and Zic are important, the necessity and sufficiency of these features have not been demonstrated. So it is possible that other inputs are also important and indeed that the two enhancers have some differences in inputs. This should be raised as a possibility and not simplified to state that these two enhancers have the same inputs and behave differently.

*This is true that the Proximal and Distal enhancers may have other inputs other than Ets and Zic factors and that not all of them may be shared between both enhancers. I don't think it changes our conclusion that these two enhancers are quantifiably different in their dependence on MAPK signaling, but we've added new language to raise this possibility as requested.*

7. Adding more to the caveats section I like the section suggesting caveats, but they should add to this that the necessary and sufficient features within the enhancer have not been experimentally determined and so these enhancers could require other tf binding sites and that there may be differences between the two enhancers and their inputs that have not yet been identified.

*This is essentially the same point as #6 and we've added this concern to the caveats section as well.*

Reviewer #3: The authors of "Ciona Brachyury proximal and distal enhancers have different FGF dose-response relationships" demonstrate that two "distributed" Brachyury regulatory elements that drive the same expression pattern behave differently in response to variations in one of the inputs (FGF/MapK signaling). The difference in response, overall, is clear, and they also attempt to ascertain if there are non-Boolean dynamics in play regarding each elements' response to MAPK interference. Furthermore, they demonstrate that native Brachyury expression is impacted similarly, and that some notochord lineages respond to MAPK inhibition differently than other notochord lineages. It is an interesting piece of work and with moderate revisions will help decipher FGF-induced transcriptional dynamics and more broadly make an important contribution towards understanding the role of "distributed" seemingly redundant enhancers in regulating gene expression.

This manuscript would be acceptable given the following issues are addressed:

1. Line 233 combined with line 243 reads as if over 16,000 individual notochord nuclei midpoints in the Z dimension were manually identified. Is that a correct interpretation? That could be made clearer and as it seems exceptionally high perhaps include reasoning as to why that many were needed (e.g. statistical robustness). The "cell measurements" excel sheet in the appendix provides a clue to what was counted (40cells x 10 embryos x # of treatments x each reporter construct) but it should be briefly mentioned.

*That is correct- we made manual measurements of over 16000 notochord cell nuclei. This was technician Julie Hix's work from home project when our lab was completely shut down due to the pandemic… It seems like an enormous number and it was indeed a lot of work, but there are 40 notochord cells in each embryo so that is only ~400 embryos split across the 3 treatments, 3 reporter constructs and 3 or more biological replicates. Given the mosaicism of expression and variability from cell to cell and embryo to embryo, this ended up being enough to draw firm conclusions but it really wasn't excessive in any way. We've added a sentence explaining these numbers.*

2. The authors state that they "only included replicates where the capacitance reported by the electroporator in time constant mode was between 900 and 1300 µFd." It would be helpful to include a description of why these parameters were used (how exceptions create unfavorable conditions/increased mosaicism if that is the case), as well as include a relevant reference.

*We don't have an ideal reference to cite here but this fits with our anecdotal experience doing extensive confocal imaging of electroporated constructs. The reasons for variable capacitance are unclear but probably have something to do with the concentration of eggs in the cuvette, the presence of lysed eggs or other debris, or something to do with how evenly the cuvette contents are mixed. We try to control for all of these things, but there remains some variability from electroporation to electroporation. Very high capacitances are very unusual in our experience. Low ones are more common and are associated with weaker and more mosaic expression. The 900 µFd cutoff is quite stringent- we usually observe reasonable expression for capacitances in the 700-900 µFd range and a more pronounced dropoff below that. The precise numbers may not have much meaning between different labs given slight differences in electroporation apparatuses and protocols. We added some new language to the manuscript to justify these parameters.*

3. Regarding the differential responses by the two promoters, what jumped out was the lack of description of ETS binding site quantities/distributions per element. Activated ETS proteins are the effective readout for FGF/MAPK signaling, and as the authors mention in the discussion the main difference in how the two promoters respond likely have to do with different quantities of ETS binding sites. It would be simple enough to generate a basic diagram of the binding sites and discuss responses versus ETS/ZicL binding site distribution.
Furthermore, hypothetically if the distal promoter had two ETS sites while the proximal had five ETS sites, hypotheses could be drawn and discussed regarding why MAPK inhibition induced a switch or fade response in the proximal or distal element.

*We think it would make more sense to leave this diagram for some future publication where we actually test mutations of different combinations of binding sites in dose-response experiments. We are excited about doing that in the future, but as we mention in the text it would really benefit from a higher-throughput reporter assay. It is probably going to be a more complicated story than just how many ETS sites there are. That would be a straightforward hypothesis, but there are actually aren't more obvious ETS sites in the proximal enhancer compared to the distal enhancer. There are several plausible mechanisms that might explain this, but I think it would make more sense to not open that bag of worms until we or others address it experimentally in the future.*

4. The description of switch vs. fade is unclear. The authors performed multiple layers of statistical analysis on their data to attempt to show that different promoters exhibited different switch vs. fade responses. But the writing surrounding the topic (the description of the results and how they were obtained), is not clear enough as it is written currently. It is entirely possible that a simple rewrite of that section will clear the matter up. What this reviewer suggests might help are descriptions of why certain data was analyzed. For example, what is it about the mean and the 90th percentile data that make it most suitable for secondary analysis? Furthermore, it should be explained to the reader how you can be sure an ON->OFF "switch" is not simply the result of a lower-expressing cell "fading" below the threshold set by the authors as ON or justify the decision use this method despite this issue. Because of the variability in TG expression patterns, it is important that the reader is guided through the information on which conclusions are drawn in order to be properly convinced.

*We've rewritten parts of this section and added new text to try to make this more clear overall and to clarify the specific points raised. This is a complicated dataset but the main concern was just to confirm that the graded responses observed when quantifying expression at the level of whole embryos are at least in part a function of expression that is truly graded at the level of single cells and not solely a function of the fraction of cells exhibiting a Boolean response.*

*We can't exclude there being some true expression in cells below the threshold we used, but that threshold was set very carefully and is essentially dependent on the background staining of the GFP antibody used. It's a very clean antibody so we are confident that we are detecting even very weak expression. We've added some qualifying language though about the possibility of very weak signal, which is generically true in just about any quantitative assay.*

5. The authors detail how image files from earlier experiments were analyzed for detection of switch vs fade mechanisms of response. However, the authors fail to mention how the actual nuclei were detected. If the fluorophores driven by each promoter were UNC76-tagged as they state, nuclear staining, if present at all, would not be more intense than the surrounding cytoplasm. This is evidenced by the images therein, as well as in all previously published reports of UNC76-tagged fluorophores. The authors must address this question.

*The nuclei are very distinctive in the phalloidin channel when viewed at full resolution. Most of the phalloidin signal is in the cell cortex but there is fainter staining in the cytoplasm.*

*The nucleus lacks that weak cytoplasmic staining and also tends to be surrounded by brighter perinuclear actin blobs. We've added some new language to make it clear that we identified nuclei based on the phalloidin channel and not the reporter channel.*

6. The authors present interesting data regarding how during gastrulation the notochord founder cells have a variable response to MAPK inhibition via U0126. Effectively, it appears the sensitivity of Bra expression to MAPK inhibition decreases moving from medial cells to the lateral cells and secondaries. Notochord cell populations can be traced back to either primary or secondary lineages (as in Corbo et al, 2006). The authors should explain why they did not choose to analyze the 2° notochord lineage (posterior cells) as a distinct population from the 1° lineage. Without any new experiments the authors should be able to provide an analysis of reporter expression in response to U0126 in the 2° notochord lineage cells (especially as the authors demonstrate that Bra expression in 2° lineage is most sensitive to U0126). This could be a valuable addition and would be simple enough to perform.

*As discussed in response to Reviewer 1, we have now taken a preliminary look at this using our cell-level quantitation at three key doses.*

7. Line 438 states that "Fertilized, dechorionated embryos were electroporated with 45μg…". This is only relevant if the reader is provided a volume of eggs + seawater electroporated.

*Good point. These were always in a total volume of 800μL and we have added that info to the methods.*

8. The authors state that it was "not feasible to split electroporated embryos between more than 8 drug treatments." It is left to the imagination as to why, as many thousands of embryos can be generated from a single transgene electroporation. This could easily be cleared up. Due to too few embryos per electroporated batch? Due to space or time constraints?

*Even with a few thousand embryos in a typical electroporation, you end up with fewer than you might think given that some fail to fertilize or develop poorly, some get lost in handling, and many end up on the slides at weird angles that aren't suitable for getting good images. The main limiting factor though was just the confocal time it took to image all the embryos in a single biological replicate. We've added a line to clarify that.*

9. Regarding the switch vs. fade section and paragraphs in the discussion, it might be helpful to include a diagram of responses, similar to Figure 3A. Figure 3A is instructive, but also misleading as it disguises the complexity of the actual situation. It would be helpful to first show a more realistic cartoon of "average" TG expression (some empty notochord cells, some with low or med or high expression, ordered essentially at random), followed by average depictions of the divergent outcomes, per TG tested. While this may not capture the completeness of the story it could be illustrative to a meaningful degree.

*This was a good suggestion. We've redone Fig 3A to incorporate mosaicism and variable expression.*

10. It is clear that the authors went to trouble to attempt to control for electroporation variation when comparing the three promoters response to concentrations of U0126. However, this reviewer wonders if there was a missed opportunity for the simple control of co-electroporating all three constructs if each TG expressed a different color. It has been demonstrated (Chen et al, 2012) that when electroporated in equimolar amounts transgenes produce consistent ratios of signal per embryo. Thus, if each Bra promoter drove a different color, the separate intensities could be compared in single embryos with only the U0126 treatment as a variable to assess, for intensity ratio of each color, per embryo, would be consistent in each electroporation.

*We agree that would be an elegant strategy with some distinct advantages. We thought about that approach when we first designed these experiments but ultimately decided against it. One concern is that the imaging ends up being more challenging in various ways if you have to worry about potential bleed between multiple reporter channels. Another is that you really need thorough tag-swapping controls to ensure that the different tags aren't contributing to the observed differences between different enhancers. At the time it seemed like this would be more cumbersome than necessary given that there seemed to be major differences in dose-response curves between the proximal and distal enhancers in preliminary proof of concept studies. As Ciona cis-regulatory analysis becomes increasingly quantitative, however, I think it is clear that multi-reporter, internally-controlled approaches will have to become more common. We used a two-color approach in (Harder et al, Dev Bio, 2019) to control for mosaicism when dissecting an enhancer specific to just the secondary notochord and it worked very well.*
